# Extensive long-distance pollen dispersal and highly outcrossed mating in historically small and disjunct populations of *Acacia woodmaniorum* (Fabaceae), a rare banded iron formation endemic

**DOI:** 10.1093/aob/mcu167

**Published:** 2014-08-06

**Authors:** Melissa A. Millar, David J. Coates, Margaret Byrne

**Affiliations:** Science and Conservation Division, Department of Parks and Wildlife, Locked Bag 104, Bentley Delivery Centre, Bentley, WA 6983, Australia

**Keywords:** *Acacia woodmaniorum*, correlated paternity, disjunct populations, dispersal distance, entomophilous pollination, gene flow, mating system, paternity analysis, pollen immigration

## Abstract

**Background and Aims:**

Understanding patterns of pollen dispersal and variation in mating systems provides insights into the evolutionary potential of plant species and how historically rare species with small disjunct populations persist over long time frames. This study aims to quantify the role of pollen dispersal and the mating system in maintaining contemporary levels of connectivity and facilitating persistence of small populations of the historically rare *Acacia woodmaniorum*.

**Methods:**

Progeny arrays of *A. woodmaniorum* were genotyped with nine polymorphic microsatellite markers. A low number of fathers contributed to seed within single pods; therefore, sampling to remove bias of correlated paternity was implemented for further analysis. Pollen immigration and mating system parameters were then assessed in eight populations of varying size and degree of isolation.

**Key Results:**

Pollen immigration into small disjunct populations was extensive (mean minimum estimate 40 % and mean maximum estimate 57 % of progeny) and dispersal occurred over large distances (≤1870m). Pollen immigration resulted in large effective population sizes and was sufficient to ensure adaptive and inbreeding connectivity in small disjunct populations. High outcrossing (mean *t*_m_ = 0·975) and a lack of apparent inbreeding suggested that a self-incompatibility mechanism is operating. Population parameters, including size and degree of geographic disjunction, were not useful predictors of pollen dispersal or components of the mating system.

**Conclusions:**

Extensive long-distance pollen dispersal and a highly outcrossed mating system are likely to play a key role in maintaining genetic diversity and limiting negative genetic effects of inbreeding and drift in small disjunct populations of *A. woodmaniorum*. It is proposed that maintenance of genetic connectivity through habitat and pollinator conservation will be a key factor in the persistence of this and other historically rare species with similar extensive long-distance pollen dispersal and highly outcrossed mating systems.

## INTRODUCTION

Patterns of pollen-mediated gene flow and variation in the mating system directly influence levels of genetic diversity, levels of genetic connectivity and genetic structure, and are key to the evolutionary potential of plant populations (Young *et al.*, 1996; Eckert *et al.*, 2010). Population genetic theory predicts disruption of genetic connectivity when populations become small and populations are fragmented or geographically isolated. A loss of allelic diversity via increased levels of genetic drift is expected to result in reduced levels of genetic diversity within populations and increased genetic divergence among populations (Slatkin, 1987; Ellstrand, 1992; Young *et al.*, 1996). Reduced population size and increased isolation may also disrupt the mating system, with reduced numbers of available mates, increased levels of selfing for self-compatible species and subsequent reduced levels of reproductive success and fitness costs to progeny via inbreeding depression (Aguilar *et al.*, 2006; Eckert *et al.*, 2010; Jacquemyn *et al.*, 2012).

Historically rare species often have naturally (i.e. non-anthropogenically induced) small effective population size, and geographically disjunct and patchily distributed populations with geographically restricted ranges (Feidler and Ahouse, 1992). In accordance with the predictions of population genetic theory, the influence of these factors on genetic connectivity is expected to be largely negative (Ellstrand, 1992; Ellstrand and Elam, 1993; Gitzendanner and Soltis, 2000). Meta analyses have shown that rare species are generally associated with low overall species diversity, low levels of within-population genetic diversity and increased levels of among-population genetic structure as a result of the heightened impacts of genetic drift under conditions of limited genetic connectivity and/or selection under a narrow range of environmental conditions (Karron, 1987; Hamrick and Godt, 1989; Gitzendanner and Soltis, 2000; Cole, 2003; Leimu *et al.*, 2006). The long-term impacts of restricted gene flow on rare species and others with small disjunct populations and geographically restricted ranges may be expected ultimately to include increased risk of extinction (Ellstrand and Elam, 1993). Despite this, many historically rare plant species with small, patchily distributed populations and geographically restricted ranges have persisted in changing environments over very long time frames, and may not exhibit the classic effects of small population theory. How well levels and patterns of genetic diversity within rare species conform to population genetic predictions of small population size will depend on a range of life history traits and ecological processes that affect pollen dispersal, and the spatial positioning of individuals at the landscape scale (Ellstrand and Elam, 1993; Hamrick and Godt, 1996; Cole, 2003; Hamrick, 2004). This makes historically rare species valuable model systems for understanding how patterns of pollen-mediated gene flow and variation in the mating system maintain genetic connectivity and genetic variation and provide for long-term persistence of populations and species. Such knowledge may also lead to increased consideration of the role of genetic connectivity in responses to more recent anthropogenic habitat disturbance, long-term adaptation and conservation, and successful restoration of remnant vegetation (Young *et al.*, 1996; Broadhurst *et al.*, 2008*a*; Eckert *et al.*, 2010; Lankau *et al.*, 2011).

Investigation of pollen-mediated genetic connectivity and variation in the mating system of rare species is readily achieved in the ancient landscape of south west Western Australia (WA) as it is rich in historically rare plant species with typically small population size and disjunct and patchy population distributions. Within this landscape, banded iron formation (BIF) inselbergs act as ecological islands that support an especially large number of edaphically endemic and historically rare species. *Acacia woodmaniorum* sect. *Alatae* is one historically rare edaphic endemic of WA's BIF outcrops that has a distribution suitable for investigation of the impacts of small disjunct populations on patterns of pollen dispersal and mating system parameters. Populations occur over two relatively large series of BIF ranges, and a number of small populations are located on small BIF outcrops at varying distances from the main ranges. The taxon was only recently described (Maslin and Buscumb, 2007), and little is known of the species biology or ecology. Seed dispersal, specific pollinators and aspects of the mating system have not been investigated.

Patterns of phylogeographic and genetic structure suggest that the distribution of *A. woodmaniorum* has been historically restricted, with long-term persistence of some small and disjunct populations along with large populations in the main range. Evidence from indirect *F*-statistic-based estimates has indicated that seed and/or pollen dispersal in *A. woodmaniorum* is sufficient to maintain a moderate degree of genetic diversity with some broad genetic structure among populations (global *F*_ST_ = 0·097; Millar *et al.*, 2013), but limited negative genetic effects of inbreeding and drift in the smallest and most spatially isolated populations (Millar *et al.*, 2013). These results led to a hypothesis that genetic connectivity, diversity and long-term persistence in this taxon are the result of extensive pollen dispersal among populations, coupled with a predominantly outcrossed mating system. Here we test this hypothesis by directly assessing contemporary patterns of pollen dispersal and other aspects of the mating system across the geographic range of *A. woodmaniorum*. We used nuclear microsatellite genotyping of progeny arrays from eight small disjunct populations for which spatial and genotypic data are already available for all adult plants. We then answered the following questions. (1) How extensive is pollen immigration into spatially disjunct, small populations? (2) Over what distances does pollen dispersal occur? (3) What are the parameters of the mating system, including rates of outcrossing, inbreeding and correlated paternity within pods? (4) Is pollen immigration, outcrossing rate or the level of inbreeding in *A. woodmaniorum* affected by population size or the degree of isolation?

## MATERIALS AND METHODS

### Study species and site

**Fig. 1.**
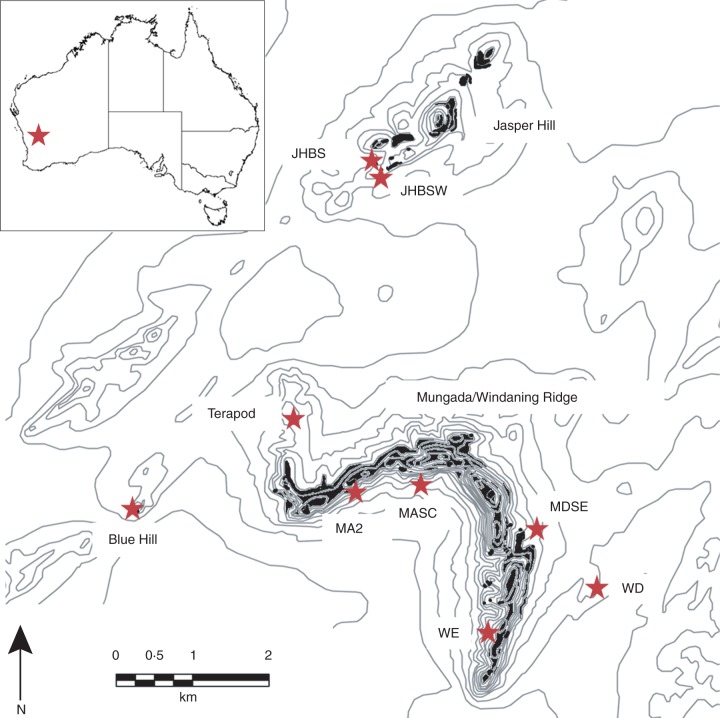
Map of the distribution (shading) and sampling locations (stars) of *Acacia woodmaniorum*. Grey lines show altitudinal contours. Population names correspond to those in Table 1.

*Acacia woodmaniorum* (Maslin and Buscomb) sect. *Alatae* (Benth.) is a sprawling, prickly, woody shrub 1–2 m tall and up to 2 m wide. Its small, globular, racemes are comprised of many light golden flowers. Pods are dark brown, narrowly oblong, curved, and sometimes twisted and 10–45 mm long. The pods bear small, hard coated seeds 3–4 mm long and dark greyish brown to black in colour, that are released following rapid dehiscence of the pods. Intensive surveillance over the species range, which covers <40 km^2^ in WA, has mapped approx. 25 000 known plants. These are highly substrate specific to the skeletal soils of the steep slopes, rock crevices and gullies of low-altitude BIF outcrops and can be broken down into two main geological and geographic regions (Fig. 1). Habitat is more or less continuous across the main range on Mungada/Windaning Ridge where the majority (approx. 18 000) of the plants occur. The Jasper Hill region to the north covers a smaller area, and populations in this region, which range in size from tens of plants to just over 1000 plants, are located on a number of smaller, discrete BIF ridges separated by intervening unsuitable habitat. Several very small disjunct populations, on which this study is focused, occur on ironstone breakaways located off these main ranges. The largest at Blue Hill region comprises 145 plants and the smallest at MDS comprises seven plants.

The midwest region where the plants occur is located approx. 330 km north north east of Perth. The region experiences a semi-desert Mediterranean climate (Beard, 1976) with annual rainfall of 300–400 mm mostly during winter. Off the BIF ranges, the surrounding clay, silt and sand plains are dominated by a low Mulga (*Acacia aneura*) woodland with other *Acacia*, *Allocasuarina*, *Melaleuca* and eucalypt species, and surrounding low hills by *Senna*, *Eremophila* and *Acacia* shrubs (Beard, 1976). The area has been subject to previous mining activity, and both the western end of Mungada Ridge and Blue Hill are highly disturbed after mining activities in the 1960s and 1970s.

### Population sampling and genotyping

In a previous study we genotyped plants from sampling locations (called populations from here on) across the species range at 15 nuclear microsatellite loci and collected detailed spatial co-ordinates with a differential Global Positioning Satellite system for each sampled plant (Millar *et al.*, 2013). Eight of these populations had varying degrees of geographic disjunction from their nearest population and nearest large population (Table 1, Fig. 1), and were treated as independent entities (populations) to test pollen dispersal and immigration. The populations were small enough such that all plants (ranging from seven to 145 plants) could be genotyped, providing circumscribed study areas for thorough paternity analysis and direct comparisons of pollen immigration rates and distances for populations of varying size and degree of isolation. For this study, we collected and genotyped progeny from the 2009 flowering season from mother plants within these eight populations. Population parameters including degree of isolation (linear distance to plants in the next closest population), degree of isolation from a large population (linear distance to plants in the next closest population containing >1000 plants) and size (the number of plants) were determined for each population (Table 1).

**Table 1.** Population size, isolation distances, number of mother plants sampled, size of progeny arrays and number of progeny used for analysis of correlated paternity, other mating system parameters and paternity for 525 progeny of *Acacia woodmaniorum*

Population	Size	Isolation (m)	Isolation from large population (m)	No. of mother plants sampled	Size of progeny arrays (= no. of pods) per mother plant	Total no. of progeny used	
						Correlated paternity analysis	Mating system and paternity analysis
JHBS	22	120	1000	3	9, 10, 10	–	29
JHBSW	17	120	980	2	6,12	–	18
MASC	8	100	170	4	7, 12, 14, 16	–	49
MDSE	7	100	450	4	16, 18, 18, 18	–	70
Terapod	10	700	940	5	1, 2, 6, 7, 9	–	25
Blue Hill	145	1870	1870	6	12, 16, 18, 19, 19, 20	–	104
WD	29	910	970	3	7, 15, 18	–	40
WE	16	180	180	4	10, 16, 18, 26	–	70
MA2				10	2, 4, 4, 4, 5, 5, 5, 6, 6, 6, 7, 7, 8, 8, 8, 8, 9, 9, 9	120	–
Total				41	525	120	405

We aimed to collect seed from ten evenly spaced maternal plants (or all plants if less than ten) within each selected population. However, pod production and seed-set was low in *A. woodmaniorum*, and in many populations few plants produced pods. In line with best practice seed collection guidelines (Mortlock, 1999), and so as not to affect population viability negatively, we harvested no more than 20 % of pods within a given population. This limited the number of maternal plants, pods and seed available for harvest, so mother plants were chosen opportunistically from those that had produced seed. The number of suitable maternal plants per population ranged from two to six (Table 1). Necessarily, mother plants in different populations occurred at various locations within populations. The mean distance between suitable mother plants within populations ranged from 3·6 to 102 m.

In *Acacia* species, the number of pollen grains in the polyad is correlated with the number of ovules in the ovary so that a single polyad pollinating a stigma is capable of fertilizing all ovules within a flower (Kenrick and Knox, 1982; Tybirk, 2007). Mating system parameters, calculated using allele frequency estimates, and patterns of pollen dispersal may be biased in cases where all seed within an *Acacia* pod are full siblings with the same paternal parent as well as the same maternal parent (Kenrick and Knox, 1982; Muona *et al*., 1991). We assessed correlated paternity within pods (*r*pp_m_) at a site within the main range (MA2; Fig. 1) so as to remove any bias from further assessment of paternity and mating system parameters. A correlated matings model was used to characterize the extent that siblings share the same father *r*_p_, the correlation of paternity. Pods were collected from ten tagged maternal plants, and all seed from two pods from each mother was sown for analysis.

Given the likelihood of a high degree of correlated paternity within *Acacia* pods, we used a sampling design that removed bias due to correlated paternity and selected a single seed from each pod for further analysis. Seed were germinated on agar in an incubator at 15 °C and then grown in potting mix under shadehouse conditions. Once seedlings had grown large enough to survive harvest, DNA was extracted from phyllodes following the methods of Millar (2009). Progeny were genotyped with a sub-set of nine previously described nuclear microsatellite markers (loci Aw124, Aw129, AwB008, AwB107, AwB108, AwC001, AwD008, AwD012 and AwD116, Millar, 2009). These loci are known to be in linkage equilibrium for adult cohorts for all populations except Blue Hill, which has undergone recent anthropogenic disturbance (Millar *et al*., 2013).

### Data analysis

#### Microsatellite variation

Samples that did not amplify clearly were re-amplified at least once. Allele bins were manually assigned and all bins and automatically assigned alleles were manually checked and adjusted when necessary. The number of alleles per locus was estimated for progeny cohorts using the GenAlEx v 6.41 program (Peakall and Smouse, 2006). Null allele frequencies were estimated for maternal plants and progeny arrays combined using the CERVUS v 3.0.3 program (Marshall *et al.*, 1998). Means and standard errors of allelic diversity statistics including the percentage of polymorphic loci, the number of alleles per locus, the number of effective alleles per locus and expected and observed heterozygosities were obtained for progeny cohorts using GenALEx.

#### Correlated paternity within pods

Seed from one pod did not survive to harvest; therefore, 19 progeny arrays comprising 120 progeny were genotyped for assessment of correlated paternity within pods. The number of progeny per pod ranged from two to nine and averaged 6·3. We used genotype data for maternal plants and progeny arrays to estimate multilocus correlated paternity, or the proportion of full sib progeny among a pair of siblings within pods (*r*pp_m_), using the sibling pair method in the MLTR v 3.4 program (Ritland and Jain, 1981; Ritland, 2002). We used the expectation–maximization algorithm and obtained the standard error with 1000 bootstraps. The effective number of pollen donors per pod was determined as *N*_e_ = 1/*r*pp_m_.

#### Pollen immigration

A total of 405 progeny collected from the eight small disjunct populations were genotyped for estimation of pollen immigration via direct paternity analysis (Table 1). The number of mother plants available for sampling (i.e. those that produced sufficient pods) per population ranged from two to six (Table 1). Given the likelihood of a high degree of correlated paternity within pods, only a single seed from a given pod was genotyped so the sizes of progeny arrays are equal to the number of pods collected from known maternal plants. The number varied from one to 26 (Table 1). Genotypic data for progeny arrays from known mother plants were combined with known genotypes of all plants in each population for direct paternity analysis. We considered all plants within each of the eight study populations as potential fathers since, within all populations, all plants were mature and presumably capable of producing pollen, excluding potentially <10 less mature plants at Blue Hill. However, inclusion of the genotypes of potentially immature plants at Blue Hill as potential parents will only act to ensure a more conservative rather than a less conservative estimate of pollen immigration.

The probability that the set of loci will exclude an unrelated candidate male parent from paternity for an arbitrary progeny when the genotype of the mother is known (average PE_2_) was determined for each population using CERVUS (Marshall *et al.*, 1998). Paternity analysis was conducted separately for each population using two programs, NEWPATXL v 5.0 and CERVUS, and the results compared. To account for the presence of null alleles and scoring error, we conducted paternity analysis for progeny with five or more loci genotyped. The NEWPATXL program uses exclusion methods to detect matches between progeny and potential paternal parents (Worthington *et al*., 1999). The significance of matches between progeny and potential paternal parents is assessed by drawing alleles at random to create pseudo-genotype files and examining levels of background paternity expected by chance. Given the degree of population isolation, the lack of data on allele frequencies in large populations, such as that occurring across Mungada/Windaning Ridge, and the likelihood of pollen immigration, we used sex-specific allele frequencies from each of the study populations for analyses. We conducted paternity analysis allowing a single mismatch between a progeny and a maternal parent, or between a progeny and a potential paternal parent, a repeat unit difference of one and a combined probability of 0·05 of a null match occurring. As the utility of the ‘repeat unit size’ function is not provided by the author, it was left at the default value. For each progeny matching the analysis criteria, we obtained a most likely father and calculated a conservative pollen immigration rate as the percentage of progeny that could not be assigned a father from within the population.

We also conducted paternity analysis with CERVUS. This program finds optimal progeny–male parent pairs and uses maximum likelihood for statistical evaluation of the matches (Marshall *et al.*, 1998). We ran the program for each population with simulation of 10 000 progeny, a known number of potential male parents (i.e. the total number of plants within the population), 100 % of potential male parents genotyped and an error rate of 1 %. Critical Delta criteria [defined as the difference in LOD (the natural log of the overall likelihood ratio) scores between the most likely paternal parent and the second most likely paternal parent] were obtained from simulations and used as a criterion for assignment of parentage. We compared trio Delta scores to assign most likely paternal parents at a strict (95 %) confidence level, a relaxed (80 %) confidence level and at <80 % confidence, and trio LOD scores to assess whether there was more than one equally likely potential father within the population (equal positive LOD scores for more than one most likely paternal parent) or whether there was no potential paternal parent within the population (i.e. a result of pollen immigration, negative LOD score). We did not allow known mothers to be potential male parents due to the high outcrossing rate. We considered all most likely fathers assigned within a population as the true most likely father, and calculated a conservative minimum pollen immigration rate as the percentage of progeny that could not be assigned any father from within the population, at any confidence level. We also calculated a maximum pollen immigration rate as the percentage of progeny that could not be assigned any father from within the population at a confidence level of <80 %.

We continued further analysis using results from CERVUS, as it provided more interpretable degrees of confidence for paternity assignments. We conducted regression analysis to test for correlation between minimum or maximum pollen immigration rates and population parameters. Because of the large number of ungenotyped potential male parent plants located across the species range, there was insufficient power to identify the specific source of pollen immigration into the disjunct small populations. To estimate pollen dispersal distances, we took a conservative approach and assumed pollen immigrated into each study population originated from the next closest population.

#### Mating system

A total of 405 progeny were genotyped for further assessment of the mating system. These were the same progeny used for paternity analysis. The number of mother plants available for sampling (i.e. those that produced sufficient pods) per population ranged from two to six (Table 1). Given the likelihood of a high degree of correlated paternity within pods, only a single seed from a given pod was genotyped so the sizes of progeny arrays are equal to the number of pods collected from known maternal plants and varied from one to 26 (Table 1).

The use of nine highly variable microsatellite loci ensures that progeny array sizes as small as two are informative in relation to mating system estimates (see Ritland and Leblanc, 2004). We used maximum likelihood methods based on the mixed mating model of Brown and Allard (1970) in MLTR to estimate the following mating system parameters for each population simultaneously: the multilocus outcrossing rate (*t*_m_), the single locus outcrossing rate (*t*_s_), the apparent level of selfing due to biparental inbreeding (*t*_m_ – *t*_s_), the correlation of selfing among maternal plants (*r*_s_) and the multilocus correlated paternity (*r*p_m_). Parameters were estimated using the expectation–maximization algorithm, with pollen and ovule gene frequencies estimated separately and standard errors obtained with 1000 bootstrap replicates, with families as the re-sampling units, and used to assess whether values were significantly different from one or from zero. Wright's fixation index was estimated for the parental (*F*_par_) and progeny (*F*_prog_) generations using GenAlEx, and the effective number of pollen donors per maternal tree was estimated as *N*_e_ = 1/*r*p_m_. Regression analysis was conducted for the outcrossing rate and the degree of biparental inbreeding, and for the outcrossing rate and population parameters.

## RESULTS

Population size was significantly positively correlated with degree of population isolation and with degree of isolation from large populations. The degree of population isolation was also significantly correlated with the degree of isolation from a large population (Table 2). The nine microsatellite markers were moderately variable within *A. woodmaniorum* progeny cohorts, with a total of 60 alleles detected in 525 seedlings. A total of 98·77 % of loci were polymorphic, the mean number of alleles per locus was 4·123 (±0·194), the mean number of effective alleles per locus was 2·070 (±0·094), and expected and observed heterozygosities were 0·445 (±0·022) and 0·490 (±0·029), respectively. Progeny allele frequencies per population are provided in Supplementary Data Table S1.

**Table 2.** Statistics of correlation analysis between population parameters for eight small disjunct populations of *Acacia woodmaniorum*

	Size			Degree of isolation		
	*R*^2^	d.f.	*P*	*R*^2^	d.f.	*P*
Degree of isolation	0·7845	7	0·0034	–	–	–
Isolation from a large population	0·6581	7	0·0145	0·8122	7	0·0143

### Correlated paternity within pods

Multilocus correlated paternity within pods calculated using the sibling pair method in MLTR was high, *r*p_m_ = 0·492 (±0·080), indicating that an average of two fathers sire all seed within pods.

### Pollen immigration

The probability that the set of loci will exclude an unrelated candidate male parent from paternity of an arbitrary progeny when the genotype of the mother is known varied for the eight populations and averaged 0·944 (Table 3). Rates of pollen immigration varied but were considerable for the eight populations when analysis was conducted with either NEWPATXL (ranging from 11·1 to 58·3 %) or CERVUS (ranging from 12·7 to 57·1 %; Table 3). Pollen immigration estimates obtained using NEWPATXL were not correlated with minimum or maximum estimates of pollen immigration obtained using CERVUS. For four populations (MASC, MDSE, Terapod and WE), there was a high level of discrimination among potential fathers within the population and little variation between minimum and maximum levels of pollen immigration determined using CERVUS. For the other four populations (JHBS, JHBSW, Blue Hill and WD), there was a degree of ambiguity regarding the most likely father and there was greater variation between minimum and maximum levels of pollen immigration for these populations. This was due to either a high proportion of progeny assignment within the stand at confidence levels <80 % (Blue Hill and WD; Table 3) or multiple potential fathers identified within the stand (JHBS and JHBSW; Table 3).

**Table 3.** Probability of exclusion (PE_2_) for nine loci and details of paternity assignments obtained using the NEWPATXL and CERVUS programs as percentages of progeny, for eight small disjunct populations of *Acacia woodmaniorum*

Population	NEWPATXL	CERVUS						
		Within-population assignment confidence					Pollen immigration	
	Pollen immigration	PE_2_	≥95 %	80–95 %	<80 %	Not identified	Minimum* pollen immigration	Maximum^†^ pollen immigration
JHBS	14·3	0·908	25·0	32·1	14·3	10·7	17·9	42·9
JHBSW	11·1	0·964	5·6	5·6	5·6	22·2	61·1	88·9
MASC	47·8	0·972	36·7	6·1	0·0	0·0	57·1	57·1
MDSE	44·9	0·936	28·6	15·7	0·0	0·0	55·7	55·7
Terapod	58·3	0·962	20·8	25·0	0·0	0·0	54·2	54·2
Blue Hill	18·8	0·954	7·8	25·5	49·0	4·9	12·7	66·7
WD	33·3	0·919	25·0	27·5	25·0	0·0	22·5	47·5
WE	42·0	0·933	30·4	27·5	1·4	0·0	40·6	42·0
Mean	33·8	0·944	22·5	20·6	11·9	4·7	40·2	56·9

The percentage of progeny with pollen sources not identified within the population is presented for NEWPATXL. For CERVUS, most likely fathers were assigned to progeny at confidence intervals of ≥95 % 80–95 %, <80 %, or, when listed as ‘not identified’, where a single most likely father was likely to exist within the population but could not be identified, i.e. more than one potential father was identified within the population, both having equal likelihoods of being the father. Values are expressed as percentages of the number of progeny analysed.

*Minimum pollen immigration estimate includes progeny which could not be assigned a most likely father within the population.

^†^Maximum pollen immigration estimate includes progeny which could not be assigned any father from within the population, at a confidence level of <80 %.

Neither the minimum nor the maximum pollen immigration rate (CERVUS) was significantly correlated with any population parameter (Table 4). Minimum estimated pollen dispersal distances into disjunct small populations were considerable, up to 1870m.

**Table 4.** Statistics of correlation analysis for minimum and maximum pollen immigration rates (CERVUS) to population parameters for eight small disjunct populations of *Acacia woodmaniorum*

Population parameter	Minimum pollen immigration			Maximum pollen immigration		
	*R*^2^	d.f.	*P*	*R*^2^	d.f.	*P*
Size	0·4410	7	0·0725	0·0508	7	0·5913
Degree of isolation	0·4008	7	0·0920	0·0099	7	0·8143
Isolation from a large population	0·3820	7	0·1024	0·1246	7	0·3912

### Mating system

The mating system was assessed in 31 progeny arrays. The number of progeny genotyped per population ranged from 18 for JHBSW to 102 for Blue Hill. Estimates of multilocus outcrossing rates and single locus outcrossing were high across all populations (Table 5). Multilocus outcrossing rates were not statistically significantly correlated with population parameters of size (*R*^2^ = 0·0372, d.f. = 7, *P* = 0·6472), degree of isolation (*R*^2^ = 0·0009, d.f. = 7, *P* = 0·9411) or isolation from a large population (*R*^2^ = 0·0546, d.f. = 7, *P* = 0·5775). Correlation of outcrossing or selfing among maternal plants (*r*_s_) and mean biparental inbreeding (*t*_m_ – *t*_s_), were low for all populations. There was no statistically significant correlation between the degree of biparental inbreeding and population parameters of size (*R*^2^ = 0·0111, d.f. = 7, *P* = 0·8047), degree of isolation (*R*^2^ = 0·0008, d.f. = 7, *P* = 0·9442) or isolation from a large population (*R*^2^ = 0·0007, d.f. = 7, *P* = 0·9505). Multilocus correlated paternity was generally low and not statistically significantly correlated with population parameters of size (*R*^2^ = 0·2022, d.f. = 7, *P* = 0·2637), degree of isolation (*R*^2^ = 0·0000, d.f. = 7, *P* = 0·9879) or isolation from a large population (*R*^2^ = 0·2196, d.f. = 7, *P* = 0·2416). Estimated numbers of pollen donors among pods within maternal trees (*N*_e_) varied (Table 5), but were equal to or far greater than the census population size for most populations. Within-population fixation indices were significantly less than zero for parental and progeny generations (Table 5).

**Table 5.** Estimates of mating system parameters for eight small disjunct populations of *Acacia woodmaniorum* including the multilocus outcrossing rate (*t*_m_), the single locus outcrossing rate (*t*_s_), the apparent level of selfing due to biparental inbreeding (*t*_m_ – *t*_s_), the correlation of selfing among maternal plants (*r*_s_), the multilocus correlated paternity (*r*p_m_), the effective number of pollen donors (*N*_e_) and Wright's fixation index for parental (*F*_par_) and progeny generations (*F*_prog_)

Population	*t*_m_	*t*_s_	*t*_m_ – *t*_s_	*r*_s_	*r*p_m_	*N*_e_	*F*_par_	*F*_prog_
JHBS	0·911 (0·047)	0·921* (0·028)	–0·010 (0·034)	0·119 (0·088)	0·022 (0·018)	45	0·000 (0·096)	–0·123 (0·102)*
JHBSW	1·000 (0·000)	0·996* (0·000)	0·004* (0·001)	0·106* (0·000)	0·000 (0·000)	∞	–0·078 (0·074)*	–0·142 (0·073)*
MASC	1·000 (0·000)	0·999* (0·000)	0·001* (0·000)	0·106 (0·085)	0·052 (0·081)	19	–0·118 (0·062)*	–0·099 (0·158)
MDSE	1·000 (0·005)	0·940* (0·020)	0·060* (0·020)	0·10 7 (0·078)	0·104 (0·081)	10	–0·186 (0·098)*	0·043 (0·053)
Terapod	1·000 (0·000)	0·991 (0·027)	0·009 (0·027)	0·110 (0·130)	0·003 (0·118)	333	0·043 (0·076)	0·062 (0·052)*
Blue Hill	0·995 (0·015)	0·968* (0·010)	0·024 (0·015)	0·0940* (0·009)	0·179* (0·044)	6	0·108 (0·080)*	0·058 (0·060)
WD	0·900* (0·000)	0·900* (0·000)	0·000 (0·000)	0·100* (0·001)	0·100* (0·001)	10	0·059 (0·107)	–0·293 (0·102)*
WE	1·000 (0·004)	1·000 (0·000)	0·000 (0·004)	0·109* (0·009)	0·035* (0·015)	16	0·017 (0·097)	–0·182 (0·067)*
Total or mean	0·975 (0·015)	0·964* (0·014)	0·011 (0·008)	0·106* (0·003)	0·062* (0·022)	16	–0·088 (0·036)*	–0·070* (0·034)

Standard errors are in parentheses.

*Values are significantly different from 1 (*t*_m_ and *t*_s_) or from zero (all other estimates).

## DISCUSSION

Contemporary estimates of pollen dispersal via paternity analysis in *Acacia woodmaniorum* were high (mean minimum estimate of 40·2 % immigration into eight small, disjunct populations) and occurred over large distances (≤1870m), confirming previous *F*_ST_-based estimates of high levels of gene flow across the species' range, including between small disjunct populations (Millar *et al.*, 2013). Predominant outcrossing and a lack of any inbreeding due to either self-pollination or mating between close relatives was a feature of the small spatially disjunct populations. Population parameters, including the size and degree of population isolation, were poor predictors of the level of pollen immigration into populations and had no influence on aspects of the mating system such as outcrossing rate, correlated paternity or biparental inbreeding, which were largely consistent across the species range. Our findings suggest that high levels of long-distance gene flow and a predominantly outcrossed mating system act to maintain large effective population sizes for small disjunct populations of *A. woodmaniorum*.

### Pollen dispersal

Estimates of pollen immigration rates and pollen dispersal distances confirmed our hypothesis that high levels of pollen-mediated gene flow maintain genetic diversity within populations and lead to marginal genetic differentiation among populations of *A. woodmaniorum* (Millar *et al.*, 2013). The proportion of pollen immigration into small, geographically disjunct populations, and the distances over which this occurs, indicates that these populations are not genetically isolated or static landscape elements and that they play an important role in maintaining genetic connectivity across the landscape. Pollen immigration rates and dispersal distances in *A. woodmaniorum* are remarkably similar to that detected for the common, widespread *Acacia saligna* where 40 % of pollen immigration into remnant patches occurred over a distance of 1650 m (Millar *et al.*, 2008, 2012). These findings contrast with general population genetic predictions for rare endemic species with small, patchily distributed populations and short ranges, which might be expected to show more limited genetic connectivity (Karron, 1987; Gitzendanner and Soltis, 2000; Byrne *et al.*, 2007; Sampson *et al.*, 2014). They are more consistent with the general patterns observed in many studies of impacts of fragmentation in tree species where extensive pollen dispersal maintains genetic connectivity (Hamrick, 2004; Kramer, 2008).

Life history traits such as large individual size and longevity tend to promote extensive pollen production and dispersal (Petit and Hampe, 2006). Individuals of *A. woodmaniorum* are shrubs of small stature, and flowering is not prolific in this species, which may be expected to limit pollen production. However, as a woody perennial, individuals are presumed to live for several decades, which typically provides more temporal opportunity for outcrossed and long-distance pollination events than in herbaceous or annual species. Patterns of extensive pollen immigration and long-distance pollen dispersal in *A. woodmaniorum* are, in fact, similar to those of a wide range of typically common and widespread, large and long-lived, temperate, neotropical and tropical tree species that maintain extensive pollen dispersal over large distances of several kilometres (Kaufman *et al.*, 1998; Nason *et al.*, 1998; White *et al.*, 2002; Dick *et al.*, 2003; Bacles *et al.*, 2005; Robledo-Arnuncio and Gil, 2005; Ward *et al.*, 2005). While extensive pollen dispersal may be expected for large, wind-pollinated forest tree species (Robledo-Arnuncio and Gil, 2005; Craft and Ashley, 2007), it may be expected to vary more for those pollinated by animals. Despite this, extensive pollen dispersal has also been identified in a range of insect-pollinated tree species (White *et al.*, 2002; Bacles *et al.*, 2005; Goto *et al.*, 2006; Byrne *et al.*, 2008; Ahmed *et al.*, 2009).

The exact mechanisms of pollen dispersal have not been studied in *A. woodmaniorum*. Although Australian *Acacia* display a range of pollen dispersal mechanisms, the nature of all *Acacia* polyads, where pollen occurs as composite units comprised of 4–32 pollen grains, means *Acacia* pollen is typically thought to be too heavy for significant wind dispersal (Kress, 1981; Kenrick and Knox, 1982). Arid zone *Acacia* species including *A. woodmaniorum* also tend to lack extrafloral nectaries that typically make those species adapted to pollen dispersal by passerine birds (Ford and Forde, 1976; Glyphis *et al.*, 1981; Knox *et al.*, 1985; Vanstone and Paton, 1988). As a result, pollen of most *Acacia* is thought to be dispersed by a range of generalist insect pollinators, including ants, moths, wasps, beetles and bees (Stone *et al.*, 2003). Generalist insects are known to be capable of affecting fat-tailed dispersal curves and long-distance pollen dispersal either directly or via pollen carryover when traversing intervening habitat matrices between plant populations (Dick *et al.*, 2003; Austerlitz *et al.*, 2004; Lander *et al.*, 2010).

Pollinator foraging and movement are likely to be influenced by a wide range of factors including the relative amount of pollen, nectar or other reward available, and hence the relative fecundity of plant populations, as well as population shape and other aspects of habitat quality (Leimu *et al.*, 2010). Pollen immigration is typically expected to decrease with increasing geographic disjunction and as populations become smaller and less dense (Aguilar *et al.*, 2006; Leimu *et al.*, 2006). Such a pattern was not observed in *A. woodmaniorum*, with population parameters being poor predictors of pollen immigration rates. This may reflect a compounding effect of a significant positive association between geographic disjunction and population size in this species, or alternatively may indicate that geographic distances between disjunct populations are not large enough to have a significant impact on the behaviour of insect pollinators. This finding may not be surprising given increasing evidence that the degree of geographic disjunction required to produce a significant level of genetic isolation between plant or tree populations may have been underestimated for a long time. An extensive literature has countered the previously held notion of fragmentation driving genetic isolation in forest tree species (Kramer, 2008; Bacles and Jump, 2011). In fact, a general pattern of negative density-dependent gene flow has been revealed for typically common, outcrossing, tree species that occur at low densities across widespread ranges (Kramer, 2008). Comparison between undisturbed and fragmented forest have revealed similar or increased levels of connectivity after fragmentation in both wind-pollinated (Robledo-Arnuncio and Gil, 2005; Craft and Ashley, 2007) and insect-pollinated species (White *et al.*, 2002; Hamrick, 2004; Bacles *et al.*, 2005; Goto *et al.*, 2006; Bacles and Ennos, 2008; Byrne *et al.*, 2008; Jha and Dick, 2010; Rosas *et al.*, 2011). This may be attributed to the characteristics of forest fragmentation that generally lead to lower conspecific density and increasing geographic extent of effective breeding units and of pollen dispersal distances (Nason *et al.*, 1998; Kramer, 2008). Long-distance dispersal means that maximum pollinator dispersal distances are not discovered in many empirical studies, and our findings suggest that maximum pollinator dispersal distances exceed 1870m for *A. woodmaniorum*.

Previous investigation of genetic structure in *A. woodmaniorum* also suggested a pattern of (presumably pollen) dispersal associated with prevailing wind conditions, indicating that generalist insects carrying pollen loads may be conveyed over long distances via thermal updrafts (Millar *et al*., 2013). Wind-mediated and directional dispersal of small insect pollinators has been documented previously over distances of tens of kilometres (Gardner and Early, 1996; Ahmed *et al.*, 2009). The potential role of wind in pollinator movement, and thus pollen dispersal, would be an interesting area of investigation for this and other endemics of terrestrial inselberg habitats, such as the BIFs of WA, and other species with outcrossed or mixed mating systems and generalist insect pollinators.

Our findings of extensive pollen immigration over large dispersal distances indicate that, like individuals of many common and widespread tree species, the disjunct, small populations of *A. woodmaniorum* are not genetically isolated. The degree of genetic connectivity produced by extensive pollen dispersal appears sufficient to provide a buffer against a low number of plants in small populations. Effective population size was greater than census population size in all but the two most isolated populations, Blue Hill and WD, which do not appear to be experiencing limited diversity in available pollen. A lack of true selfing, little evidence of biparental inbreeding and low to negative values of the fixation index suggest that virtually all effective mating in *A. woodmaniorum* occurred between genetically unrelated plants and pollen dispersal is sufficient to produce ‘inbreeding connectivity’ (Lowe and Allendorf, 2010), largely limiting any negative genetic effects of inbreeding due to direct mate limitation, in even the smallest disjunct populations.

### Mating system

*Acacia* display a wide range of both asexual (Coates, 1988; NSWNPWS, 2003) and sexual mating systems, with sexual mating systems that vary from predominantly outcrossing (see Philip and Sherry, 1946; Moffet, 1956; Bernhardt *et al.*, 1984; Moran *et al.*, 1989*a*; Muona *et al.*, 1991; Broadhurst *et al.*, 2008*b*; George *et al.*, 2008; Millar *et al.*, 2008; Ng *et al.*, 2009), to substantial levels of selfing (Mandal *et al.*, 1994; Coates *et al.*, 2006). All members of the predominant Australian subgenus *Phyllodineae*, including *A. woodmaniorum*, have protogynous flowers however, a mechanism that promotes outcrossing, although there can still be great variation in outcrossing rates among populations of a single species (Coates, 1988; Mandal and Ennos, 1995). The high outcrossing rates and lack of true selfing found in *A. woodmaniorum* are comparable with those obtained from genetic studies of a number of other *Acacia* species (Moran *et al.*, 1989*b*; Casiva *et al.*, 2004; Broadhurst *et al.*, 2008*b*; Millar *et al.*, 2008), and demographic studies of seed-set indicate that many *Acacia* are either highly self-incompatible, or at least partially self-incompatible (Kenrick and Knox, 1989; Morgan *et al.*, 2002). Estimates of mating system parameters were remarkably consistent across all populations of *A. woodmaniorum* and did not vary with population parameters of size and isolation, providing further support for a self-incompatible mating system.

Despite its rarity, short range and persistence in small populations, *A woodmaniorum* shows high outcrossing and a self-incompatible mating system. Long-lived woody perennial tree and shrub species tend to have higher genetic loads, resulting in strong inbreeding depression and, hence, tend to be self-incompatible (Petit and Hampe, 2006). Self-incompatibility can be explained by pre-zygotic stylar incompatibility and/or post-zygotic seed abortion mechanisms. Confirmation of pre- or post-zygotic self-incompatibility mechanisms in *A. woodmaniorum* would require assessment of the success of controlled crosses. Field observations did indicate very low levels of pod- and seed-set over the species range despite high levels of seed-set in many other sympatric species in the year of sampling (M. Millar, DPAW, Perth, Australia, unpubl. res.). This observation suggests that poor pod-and seed-set in *A. woodmaniorum* was not solely a result of adverse temporal environmental conditions and, combined with high levels of abortion of developing pods and seed (M. Millar, DPAW, Perth, Australia, unpubl. res.), may indicate post-zygotic seed abortion or the effect of inbreeding depression following self-pollination or when pollination occurs between related individuals.

The realized outcrossing rate obtained here will be biased by any inbreeding depression resulting in seed abortion after fertilization as well as that operating on young seedlings arising from initially viable seed. Inbreeding depression could be further quantified in this species with controlled crossing experiments. The negative demographic impacts of mate limitation and reduced connectivity have recently been shown to be especially evident in self-incompatible species, although our findings suggest that populations of *A. woodmaniorum* are not mate limited due to extensive pollen dispersal (Aguilar *et al.*, 2006; Honnay and Jacquemyn, 2007; Leimu *et al.*, 2010). Analysis of levels of recruitment and long-term demographic response in populations of *A. woodmaniorum* would also be valuable in providing further insight into minimum seed production required for population persistence.

### Conclusions

Maintenance of genetic connectivity through significant pollen-mediated gene flow over extensive dispersal distances, and high levels of outcrossing, are important features of *A. woodmaniorum* that may be critical for the persistence of this species in a series of large and small disjunct populations over a narrow geographic range. As long as this population system remains intact, this species is likely to persist, even as small populations, over significant historical time frames (Millar *et al*., 2013). *Acacia woodmaniorum* is currently a listed threatened species under the Western Australian Wildlife Conservation Act 1950 (see http://florabase.dpaw.wa.gov.au), due to its highly restricted distribution and the prospective mineral exploration and active extraction activities that cover its range. Future anthropogenic disturbance is also likely in this landscape, and loss of populations may impact gene flow patterns and thus influence population persistence. A number of conservation measures that aim to alleviate negative genetic and demographic impacts of reduced connectivity can be employed for the long-term conservation of recently fragmented species and those for which further or future population fragmentation is envisaged. Maintenance of gene flow can be achieved by the direct augmentation of populations or establishment of populations at previous or new sites with germplasm sourced from a number of different populations. Genetic augmentation is likely to improve mate availability and reproductive output, but must also take into account the likelihood of any fitness reduction via outbreeding depression in the resulting progeny (Byrne *et al.*, 2011; Weeks *et al.*, 2011). Adaptation to different environmental conditions is unlikely for *A. woodmaniorum* given the habitat specificity and limited geographic range, and this, in combination with a highly outcrossed mating system, suggests that outbreeding depression is unlikely to be an issue. Levels of genetic diversity (Millar *et al*., 2013) and a lack of inbreeding effects in small populations imply that direct genetic rescue is not immediately required in this species as long as mate limitation or limitations to pollen dispersal remain minimal.

## SUPPLEMENTARY DATA

Supplementary data are available online at www.aob.oxfordjournals.org and consist of Table S1: Allele frequencies of nine nuclear microsatellite loci in progeny cohorts from nine populations of *Acacia woodmaniorum*.

Supplementary Data
