# Supplementary material for: Extensive long-distance pollen dispersal and highly outcrossed mating in historically small and disjunct populations of Acacia woodmaniorum (Fabaceae), a rare banded iron formation endemic
Source: Ann Bot. 2014 Aug 6;114(5):961–71. doi: 10.1093/aob/mcu167 (PMC4171076; doi:10.1093/aob/mcu167)
Supplement: Supplementary Data [file supp_114_5_961__index.html]

Extensive long-distance pollen dispersal and highly outcrossed mating in historically small and disjunct populations of Acacia woodmaniorum (Fabaceae), a rare banded iron formation endemic — Supplementary Data 

# Extensive long-distance pollen dispersal and highly outcrossed mating in historically small and disjunct populations of *Acacia woodmaniorum* (Fabaceae), a rare banded iron formation endemic

## Supplementary Data

Supplementary Data

**Files in this Data Supplement:**

- Supplementary Data - Pdf file
