# Supplementary material for: Extensive long-distance pollen dispersal and highly outcrossed mating in historically small and disjunct populations of Acacia woodmaniorum (Fabaceae), a rare banded iron formation endemic
Source: Ann Bot. 2014 Aug 6;114(5):961–71. doi: 10.1093/aob/mcu167 (PMC4171076; doi:10.1093/aob/mcu167)
Supplement: Supplementary Data [file supp_mcu167_mcu167supp.pdf]

SUPPLEMENTARY DATA

Table S1. Allele frequencies of nine nuclear microsatellite loci in progeny cohorts from nine populations of *Acacia woodmaniorum*.

		Population								
Locus	Allele/n	JHBS	JHBSW	MASC	MDSE	Terapod	Blue Hill	WD	WE	MA2
A129	288	0.000	0.000	0.000	0.000	0.000	0.005	0.000	0.000	0.000
	290	0.000	0.000	0.011	0.000	0.000	0.005	0.000	0.000	0.000
	294	0.232	0.029	0.096	0.000	0.125	0.022	0.000	0.000	0.014
	296	0.179	0.294	0.021	0.362	0.063	0.022	0.000	0.080	0.267
	298	0.589	0.676	0.872	0.623	0.729	0.923	1.000	0.920	0.695
	301	0.000	0.000	0.000	0.014	0.000	0.000	0.000	0.000	0.000
	302	0.000	0.000	0.000	0.000	0.042	0.016	0.000	0.000	0.019
	304	0.000	0.000	0.000	0.000	0.042	0.005	0.000	0.000	0.005
A124	185	0.000	0.000	0.000	0.000	0.000	0.015	0.000	0.000	0.000
	190	0.154	0.000	0.031	0.081	0.239	0.136	0.000	0.080	0.087
	192	0.635	0.917	0.510	0.110	0.587	0.646	0.313	0.783	0.582
	194	0.135	0.056	0.163	0.294	0.022	0.106	0.213	0.022	0.120
	196	0.077	0.028	0.133	0.272	0.152	0.096	0.475	0.116	0.082
	198	0.000	0.000	0.000	0.029	0.000	0.000	0.000	0.000	0.067
	199	0.000	0.000	0.000	0.213	0.000	0.000	0.000	0.000	0.005
	200	0.000	0.000	0.020	0.000	0.000	0.000	0.000	0.000	0.058
	201	0.000	0.000	0.143	0.000	0.000	0.000	0.000	0.000	0.000
B008	278	0.071	0.000	0.000	0.000	0.000	0.000	0.000	0.000	0.048
	281	0.000	0.000	0.000	0.000	0.000	0.010	0.000	0.000	0.000
	286	0.000	0.000	0.000	0.000	0.000	0.005	0.000	0.007	0.000
	288	0.000	0.000	0.240	0.007	0.196	0.115	0.115	0.007	0.087
	290	0.839	0.912	0.594	0.841	0.761	0.719	0.679	0.662	0.587
	291	0.000	0.000	0.000	0.058	0.043	0.042	0.000	0.000	0.000
	294	0.089	0.088	0.156	0.000	0.000	0.047	0.013	0.096	0.279
	295	0.000	0.000	0.010	0.094	0.000	0.063	0.192	0.228	0.000
D116	145	0.000	0.000	0.000	0.022	0.000	0.021	0.000	0.007	0.000
	146	0.036	0.000	0.000	0.000	0.000	0.000	0.000	0.000	0.000
	148	0.000	0.000	0.000	0.015	0.000	0.000	0.000	0.000	0.000
	151	0.732	0.972	0.980	0.838	0.688	0.649	0.400	0.979	0.788
	154	0.232	0.028	0.020	0.125	0.313	0.325	0.600	0.014	0.212
	156	0.000	0.000	0.000	0.000	0.000	0.005	0.000	0.000	0.000
B108	207	0.000	0.000	0.000	0.000	0.000	0.000	0.013	0.000	0.004
	209	0.000	0.000	0.000	0.000	0.021	0.005	0.000	0.000	0.000
	211	0.034	0.000	0.000	0.000	0.000	0.000	0.000	0.000	0.021
	213	0.086	0.000	0.378	0.136	0.792	0.639	0.218	0.529	0.701
	215	0.000	0.000	0.092	0.000	0.021	0.019	0.000	0.000	0.038
	217	0.000	0.111	0.020	0.143	0.000	0.014	0.590	0.029	0.004
	219	0.000	0.000	0.265	0.100	0.000	0.010	0.154	0.058	0.009
	221	0.000	0.000	0.000	0.000	0.000	0.000	0.000	0.058	0.000
	228	0.052	0.194	0.000	0.014	0.000	0.188	0.026	0.304	0.064
	229	0.000	0.000	0.000	0.007	0.000	0.000	0.000	0.000	0.000
	230	0.776	0.667	0.133	0.579	0.125	0.115	0.000	0.022	0.085

	232	0.052	0.028	0.000	0.021	0.000	0.010	0.000	0.000	0.013
	234	0.000	0.000	0.112	0.000	0.042	0.000	0.000	0.000	0.060
C001	142	0.000	0.000	0.000	0.000	0.000	0.000	0.000	0.000	0.004
	145	0.000	0.000	0.000	0.000	0.000	0.000	0.000	0.000	0.021
	146	0.000	0.000	0.112	0.000	0.060	0.091	0.000	0.044	0.000
	152	0.018	0.083	0.020	0.079	0.040	0.058	0.050	0.081	0.000
	153	0.000	0.000	0.000	0.000	0.000	0.000	0.000	0.000	0.126
	156	0.518	0.556	0.582	0.814	0.820	0.736	0.625	0.515	0.761
	159	0.464	0.361	0.286	0.107	0.080	0.115	0.325	0.360	0.088
D008	187	0.000	0.000	0.000	0.000	0.000	0.021	0.000	0.000	0.009
	193	0.019	0.000	0.439	0.029	0.571	0.515	0.088	0.486	0.306
	195	0.231	0.294	0.337	0.100	0.167	0.191	0.138	0.065	0.243
	198	0.000	0.000	0.000	0.000	0.048	0.000	0.000	0.000	0.000
	202	0.000	0.000	0.000	0.007	0.000	0.000	0.000	0.000	0.000
	206	0.000	0.000	0.000	0.000	0.000	0.000	0.000	0.000	0.005
	209	0.750	0.706	0.224	0.864	0.214	0.273	0.775	0.449	0.437
B107	123	0.000	0.000	0.000	0.000	0.000	0.000	0.000	0.000	0.005
	191	0.000	0.000	0.000	0.015	0.000	0.000	0.000	0.000	0.000
	197	0.241	0.139	0.010	0.522	0.136	0.127	0.175	0.485	0.204
	199	0.000	0.000	0.000	0.000	0.045	0.127	0.000	0.000	0.000
	203	0.241	0.000	0.408	0.015	0.091	0.333	0.550	0.088	0.134
	205	0.000	0.000	0.306	0.000	0.000	0.005	0.000	0.000	0.009
	209	0.000	0.000	0.000	0.000	0.000	0.000	0.000	0.000	0.005
	211	0.086	0.083	0.082	0.294	0.045	0.029	0.000	0.147	0.264
	213	0.017	0.028	0.133	0.007	0.250	0.118	0.063	0.081	0.204
	214	0.000	0.000	0.000	0.000	0.000	0.000	0.000	0.007	0.000
	215	0.069	0.139	0.000	0.000	0.000	0.010	0.000	0.000	0.000
	217	0.345	0.611	0.000	0.147	0.432	0.250	0.213	0.191	0.176
	219	0.000	0.000	0.061	0.000	0.000	0.000	0.000	0.000	0.000
D012	233	0.036	0.029	0.426	0.050	0.130	0.130	0.474	0.130	0.035
	234	0.000	0.000	0.000	0.050	0.022	0.010	0.000	0.000	0.000
	236	0.000	0.000	0.000	0.000	0.000	0.015	0.000	0.000	0.017
	238	0.000	0.000	0.000	0.000	0.000	0.000	0.000	0.000	0.009
	239	0.964	0.971	0.574	0.900	0.848	0.845	0.526	0.870	0.939
